# Endomyocardial Biopsy in Pediatric Myocarditis and Dilated Cardiomyopathy: A Tool in Search for a Role

**DOI:** 10.3390/jcdd9010024

**Published:** 2022-01-12

**Authors:** Mara Pilati, Micol Rebonato, Roberto Formigari, Gianfranco Butera

**Affiliations:** Medical and Surgical Department of Pediatric Cardiology, Ospedale Pediatrico Bambino Gesù, IRCCS, Piazza di Sant’Onofrio 4, 00165 Rome, Italy; Micol.rebonato@opbg.net (M.R.); roberto.formigari@opbg.net (R.F.); gianfranco.butera@opbg.net (G.B.)

**Keywords:** endomyocardial biopsy, myocarditis, pediatric cardiomyopathy

## Abstract

Endomyocardial biopsy (EMB) is a well-known diagnostic tool for the investigation and treatment of myocardial diseases and remains the gold standard for the diagnosis of myocarditis. Due to its invasiveness, with a complication rate ranging from 1 to 15%, its role in the diagnostic work-up of pediatric heart failure is not well established. The aim of this review is to define the role of EMB as diagnostic technique in the work up of children presenting with severe left ventricular dysfunction with the support of our center experience.

## 1. Introduction

A child presenting with systolic left and right ventricular dysfunction is always a challenging scenario for clinicians in terms of diagnosis, therapeutic management, and follow up. 

Pediatric heart failure shares common features with that of the adult population but with important different characteristics and etiologies, making the use of adult guidelines not very useful.

Etiology particularly has great relevance in terms of prognosis. Depending on the etiology, the clinical outcomes of children with cardiomyopathies are strongly variable, with the rate of 5-year transplant-free survival ranging from 20% to 94% [1]. Infants with idiopathic dilated cardiomyopathy have a higher risk of death or heart transplantation than those with other causes of DCM [2].

The identification of the causes of DCM in children is very difficult, and a standardized approach to a diagnostic work up still does not exist. 

Ecocardiography is the first step and is the most helpful instrument for the evaluation of LV function and for the identification of the most common causes of HF, such as valvular disease, congenital heart disease, or other forms of primary cardiomyopathies. 

In recent decades, other noninvasive tests have been integrated in the diagnostic work up for the management of pediatric heart failure. 

Since the first report of its use in children with systolic LV dysfunction [3], CMR has gained a widespread use. A report from the Pediatric Health Information System (PHIS) database showed a five-fold increase (5.2% to 28.1%) in the use of CMR in children with myocarditis between 2006 and 2011 [4].

Endomyocardial biopsy (EMB) is a well-known diagnostic instrument and remains the gold standard for the diagnosis of myocarditis. Histological, immunohistochemical, and virological studies on EMB specimens allow the characterization of immune cell infiltrates, the detection of viral RNA and DNA, and the quantification of viral load. 

Prospective studies describing the utility and EMB-related risks are lacking, so its role in the work up of children with LV dysfunction is still debated. 

In 2007, the American Heart Association (AHA), the American College of Cardiology, and the European Society of Cardiology (ESC) published a scientific statement on the use of EMB [5]. The consensus statement outlined the scarcity of published data about the efficacy and safety of EMB in children, so physicians must count on their clinical judgement when deciding to perform a cardiac biopsy.

The aim of this review is to define the role of EMB as a diagnostic tool in the management of infants presenting with severe left ventricular dysfunction with the support of our center experience.

## 2. EMB: How and When It Should Be Performed

Cardiac biopsy was introduced as a diagnostic instrument in the 1950s, when myocardial tissue from humans was obtained through a transthoracic needle approach [6].

In the 1960s, the safety of the heart biopsy procedure improved after the introduction of vascular access and X-rays that allowed samples to be taken from the right ventricular septum [7]. The use of a flexible bioptome with sharpened cusps was introduced by Sakakibara and Konno in 1962 [8]. Konno biopsy forces were then modified by Caves et al. [9] in order to perform the procedure through the internal jugular vein, and the Stanford–Caves bioptome is still used for EMB.

The right internal jugular vein is the most common percutaneous access point to the right ventricular EMB in children, allowing for the easy access to the right ventricle. The bioptome can be inserted through a sheath that is at least 6 Fr. Biopsy of left ventricle via the femoral artery or via transseptal puncture are also described but are rarely used in children, with only 19 cases being published in 1984 [10].

Usually, EMB in children is performed under general anesthesia because of their young age but also because these patients frequently present with hemodynamic instability. EMB is usually performed under fluoroscopic guidance. Fluoroscopy is generally better than two-dimensional echocardiography in terms of guiding the entry of the bioptome in the right ventricle and in terms of determining the correct position for the biopsy [11]. Some operators use fluoroscopy and echocardiography together in order to better define the EMB site [12].

EMB is a cardiac interventional procedure that is used to guide the diagnosis but also to guide the prognosis and management of cardiomyopathies. It is considered the gold standard in the diagnosis of myocarditis, but the real indication in pediatric population is still controversial. Prospective studies describing the utility of EMB in the pediatric population are lacking, so current recommendations rely exclusively on case–control series and expert consensus. 

The recently published ESC guidelines for acute and chronic heart failure [13] do not take into account the pediatric population and state that in adults with heart failure, EMB should be performed in patients with rapidly progressive HF despite standard medical therapy when there is a high suspicion of a specific disease, with a class IIa C-level of evidence. 

The recently published consensus document on the management of acute myocarditis and chronic inflammatory cardiomyopathy [14] states the role of EMB for the etiopathology identification and therapy decision in specific clinical scenarios:

1. Acute myocarditis (AM) presenting with severe HF or cardiogenic shock;
2. AM associated with severe myocardial dysfunction, acute HF, ventricular arrhythmias, or high degree atrio-ventricular block;
3. AM or suspected chronic inflammatory cardiomyopathies (infl-CMP) presenting with peripheral eosinophilia;
4. AM or chronic inf-CMP in the presence of a persistent or relapsing release of myocardial necrosis biomarkers, particularly if there is a suspected/known autoimmune disorder or ventricular arrhythmias or high degree AV block;
5. Myocarditis in patients treated with immunotherapy, where appropriate diagnosis has implications in additional cancer therapy.

Most of these recommendations were first published in the 2007 AHA/ACC/ESC Scientific Statement on the role of EMB in the management of cardiovascular disease [15].

In this statement, EMB was recommended for unexplained cardiomyopathy (secondary forms excluded) in children presenting with hemodynamic compromise or arrhythmias. 

The exact timing of EMB for a useful recognition of the etiology of unexplained LV dysfunction in children is also controversial. The diagnostic yield of EMB is considered to be higher if performed within 2 weeks since the onset of symptoms, and it is usually better to perform EMB when a period of hemodynamic stabilization (normally 24 h) is achieved and within at least 72 h of hospital admission [16].

Infants with myocarditis can often go into cardiogenic shock, and the use of mechanical circulatory support (MCS), such as extracorporeal membrane oxygenation (ECMO) or ventricular assist devices, is needed. EMB can also be undertaken during ECMO placement.

## 3. EMB: Which Kind of Information

One diagnostic limitation of EMB are sampling errors that may occur due to the patchy position of the disease. In order to minimize sampling errors, a large number of pieces (usually >5) must be collected and handled carefully. The number of specimens is usually determined by the clinical reason for EMB. Usually, 4–5 samples are used for light microscopy, but more may be submitted for electron microscopy.

Some myocardial samples, preferably four, should be fixed in 10% buffered formalin for light microscopic examination; one or two specimens may be frozen in liquid nitrogen and stored at −80 degrees for molecular tests, and, if ultrastructural tests are needed, then one fragment should be fixed in 2.5% glutaraldehyde.

EMB specimens are analyzed in order to produce histologic information about the chrematistics of the cardiomyocytes and of the interstitial tissue, to assess the type of inflammatory infiltration, and to detect the viral genome. 

A 2013 ESC position paper outlined the importance of the characterization of cardiac inflammation utilizing immunohistochemistry and viral genome analysis with quantitative PCR (real-time PCR and nested PCR with reverse transcription) for myocarditis diagnosis and for the use of specific therapeutic treatments [17].

Histological analysis can provide information about the characteristics of the endocardium, myocardium, and interstitium. Most of the important findings are hypertrophy, which is derived from the myocardial cell diameter, nuclear arrangement, interstitial fibrosis, the presence of inflammatory cells, fat infiltration, endocardial thickening, which is typical of endocardial fibroelastosis, and the state of the intramural microvascular arterioles [18]. 

Transmission electron microscopy is useful for the detection of infiltration in suspected infiltrative disorders and in anthracycline toxicity [15].

Immunohistochemistry can better define the nature of infiltrative cells. The use of specific antibodies for leukocytes (CD 45), macrophages (CD 68), T cells (CD3) and their main subtypes, helper (CD4) and cytotoxic (CD8) cells, and B cells (CD19/CD20) increases the sensitivity of EMB [17]. 

In the past, according to the Dallas criteria, the diagnosis of myocarditis was only based on the characterization of inflammatory infiltrates, and the quantification of infiltrates was not recommended [19]. 

A study among adult patients with a clinical suspicion of myocarditis showed that the use of the histopathological Dallas criteria alone only predicted inflammation in 38% of the biopsies, and additional immunohistopathological studies increased the diagnostic power of EMB [20]. In order to enhance the sensitivity of EMB, a position paper by ESC experts [17] recommended the use of the Marburg criteria in the diagnostic work up of myocarditis. The presence of >14 mononuclear leukocytes/mm^2^ with the presence of >7 T lymphocytes/mm^2^ on bioptic samples is highly suggestive of myocarditis [21,22].

Quantitative PCR allows the detection of viral RNA and DNA, the quantification of viral load, and the recognition of virus subtypes via sequencing techniques [23]. Frozen EMB tissue, as opposed to fixed tissue, is useful when using PCR to analyze the viral genome. The use of PCR and reverse transcriptase PCR (rtPCR) techniques can identify the presence of less than 10 copies of viruses in the myocardium [24].

Over the past two decades, the use of nested PCR has substantially improved the search for cardiotropic viruses in patients with inflammatory cardiomyopathy. 

Many studies of patients with myocarditis or dilated cardiomyopathy have found plenty of viral pathogens such as enteroviruses, adenoviruses, parvovirus B19, cytomegalovirus, influenza and respiratory syncytial virus, herpes simplex virus, Epstein–Barr virus, human herpes virus 6, HIV, and hepatitis C [25,26]. Previously in the United States, entero and adenoviruses were recognized as the most common causes of viral myocarditis, but in recent years, the tendency has changed, with parvovirus B 19 and herpes virus being the most common isolated viruses [27]. 

An important study from Kuhl et al. [28] showed that genomes from cardiotropic viruses can be detected in the EMBs of 75% of patients with suspected myocarditis. Parvovirus B19 and HHV6 were the more frequently detected viruses, with an incidence of 70% and 14–18%, respectively.

## 4. Complications

Acute biopsy complications include ventricular perforation with subsequent pericardial tamponade, ventricular or supraventricular arrhythmias, heart block, pneumothorax, the puncture of central arteries, pulmonary embolism, nerve paresis, venous hematoma, and damage to the tricuspid valve. In some cases, complications can be delayed and can include access site bleeding, damage to the tricuspid valve, pericardial tamponade, and deep venous thrombosis [5]. 

The data on EMB risks are derived from several single-center experiences and registries reported in literature, but the majority of these reports only refer to heart transplant patients.

In 1999, Pophal et al. [29] analyzed data from 150 children with cardiomyopathy who had undergone EMB in a population of 1000 total pediatric bioptic procedures. An analysis of the non-transplant population showed a 9% of incidence of serious complications, whereas when transplant cohort was included, the incidence was 2%. Younger age, smaller size, inotrope use, and suspected myocarditis were the risk factors associated with cardiac tamponade during EMB.

Similar data were found by Zhorne et al. [30], who reported on 99 EMBs: 49 in patients with suspected myocarditis, 43 in post-transplant patients, 3 to identify tumor histology, and 4 in patients with suspected endocardial fibroelastosis. They described 12 (12.1%) complications: nine arrhythmias and four perforations requiring pericardiocentesis. During univariate analysis, body weight < 8 kg and age < 6 months were strongly related to complications.

In 2016, two multicenter studies on EMB complications in children with suspected myocarditis were published. Brighenti et al. [31] analyzed the results of EMBS in an Italian pediatric population. Over a period of two years, 45 EMBs were performed. The overall incidence of EMB-related complications was 15.5% (31.2% in infants and 6.8% in children > 1 year); they counted one case of severe tricuspid damage, three cases of cardiac chamber perforations, one case of ventricular fibrillation, one case of conduction disturbance, and one non-specified minor complication. Cardiac tamponade due to heart perforation only occurred in infants < 1 year of age.

In the same year, Mills at al. [32] reported their data about EMBs performed in seven institutions that were part of the Congenital Cardiac Catheterization Outcomes Project. The cohort included children with cardiomyopathies (158 pts) and in children who had received heart transplants (2665 pts). Within the cardiomyopathy group, 16 total adverse events (10% of EMB cases) occurred, which was three times the rate described in the post-transplant patients. Eight adverse events were defined as being highly severe and were described in 5% of the cardiomyopathy EMB cases, while only 1.1% were observed in the transplanted population. Major complications included one myocardial perforation, two ECMO cannulations, and three deaths. Factors that were found to be associated with a high-risk of adverse events included an associated catheter-based intervention and longer fluoroscopy time. The most common additional procedure in the study was the creation or dilatation of an atrial septal defect in order to decompress the left atrium. When EMB was the only intervention, no complications occurred.

Cardiac perforation is undoubtedly of major concern when assessing the risk of performing EMBs in infants. All of the reports recognized young age and low weight as the most important risk factors. Others hypothesized that cardiac perforation may be due to ventricular walls thinness in patients with DCM. Nevertheless, this hypothesis was not confirmed in a large adult EMB series, where all of the perforations occurred in patients without ventricular dilatation. [33].

Surely, the risks and benefits of EMB should be carefully weighed in small infants weighing less than 8 Kg. Given the high risk of cardiac perforation in this population, the use of echocardiographic guidance during the procedure in order to confirm bioptome position could be useful, as demonstrated in the study of McCreery et al. [34].

## 5. Our Experience

We performed a retrospective review of the data from children who underwent endomyocardial biopsy at our Center from 2011 to 2021. Patients who had undergone heart transplantation procedures were excluded from this analysis.

During the study period, 29 children underwent 30 EMBs for systolic LV dysfunction. The characteristics of the population are described in Table 1. The mean age of the patients was 7.3 year, five children (17%) were <1 year old, and two of them weighed < 8 kg.

At admission, 24 pts (83%) presented with heart failure, 1 presented with AV block, 2 presented with pericarditis, 1 presented with abdominal pain, one child presented with COVID infection, and one presented with an isolated right ventricular dysfunction. 

In this cohort of children with a clinical suspicion of myocarditis, the histopathological analyses of the myocardial specimens provided heterogeneous results (Table 2). 

A total of 17 patients (57%) had a diagnosis of myocarditis, 9 patients received a diagnosis of primary dilated cardiomyopathy, 1 child had carnitine deficiency, 1 was diagnosed with a laminopathy, and the patient whose presentation was an isolated right ventricular dysfunction had Uhl disease (post-mortem finding).

Viral PCR on the myocardial specimens was positive in 12/17 patients (70%), and viral myocarditis was confirmed. A total of 10 patients had copies of the PVB19 viral genome (83%), 1 patient had copies of the HHV6 viral genome, and 1 patient had copies of the influenza A viral genome.

Twelve patients underwent cardiac MRI before EMB. In five patients, the MRI was positive for myocarditis, and three patients received an EMB diagnostic for acute myocarditis. Among the seven patients whose MRIs were negative for the presence of acute inflammation, three had an EMB that was positive for myocarditis.

The EMB results led to a change in medical therapy in 12 patients (40%). Treatment changes included antiviral therapy in five patients with viral myocarditis, immunoglobulin therapy in six patients, carnitine supplementation in one patient, and immunosuppressive therapy in one patient with virus negative myocarditis. 

Regarding complications, cardiac perforation occurred in three patients (10%). The first patient, who weighed 20 kg and who had EMB-proven myocarditis, underwent a pericardiocentesis and experienced almost complete recovery of left ventricular function after one month. The second, who weighed 10.5 kg, started to bleed 3 h after the procedure during thrombolytic infusion for a femoral artery occlusion. Additionally, this patient experienced the complete recovery of left ventricular function. The third patient, who weighed 6 kg, presented with isolated right ventricular dysfunction and marked thinness of the right ventricular wall. He died one week after the procedure due clinical deterioration even though he received ECMO implantation.

## 6. EMB in Myocarditis

The true incidence of myocarditis in the pediatric population is uncertain. The National Australian Childhood Cardiomyopathy Study showed that children presenting with dilated cardiomyopathy (DCM) had an incidence of lymphocytic myocarditis in 36% of cases [35], and almost identical results were found when analyzing explanted hearts after heart transplantation [2,36].

The diagnosis of myocarditis is a complex clinical process, and EMB that fulfills the well-known histologic criteria is still the gold-standard. Although imaging techniques such as MRI can determine tissue characterization and can localize areas of inflammation or identify the presence of fibrosis, they cannot totally substitute EMB due to the high negative predictive value [37]. According to a recently published report of pooled data from control trials, the diagnostic accuracy of MRI in detecting myocardial inflammation is 68–78% [38]. Moreover, MRI cannot define the characteristics of infiltrated immune cell subtypes or the numbers of inflammatory cell; for example, it is unable to quantify different virus types and loads [39]. A recently published meta-analysis [40] compared MRI with EMB for the diagnosis of myocarditis. Compared to EMB, MRI seemed to have moderate accuracy in the diagnosis of acute or chronic myocarditis. The authors concluded the necessity to develop novel MRI-related sequences and novel imaging techniques for the diagnosis of myocarditis. 

EMB has not only been used for diagnostic purpose but also can have prognostic implications and can guide therapy.

As demonstrated in some studies, no differences have been detected in long-term outcomes regardless, of the presence or absence of myocardial inflammation in patients with acute-onset LV dysfunction [41]. A study from the Pediatric Cardiomyopathy Registry [2] showed that patients with myocarditis have a better outcome than those with idiopathic DCM. The same study found no differences in terms of death, transplantation, and echocardiographic normalization between children with EMB-proven myocarditis and children with probable myocarditis. 

Gagliardi et al. [42] analyzed the impact of the presence of viruses on EMB in a large population of children presenting with acute heart failure. The presence of viruses was detected in 30% of the population, and PVB19 was the most frequently isolated virus. In the group of patients with myocarditis, the incidence of adverse cardiac events was significantly lower compared to patients without myocarditis. On the basis of virus presence, virus-positive PCR patients presented with a higher rate of freedom from adverse events when compared to those with a virus-negative PCR.

Pietra et al. [43] assessed early predictors of survival in children with DCM up to and after heart transplantation (HT). They stated that children with myocarditis at presentation have an increased risk of death after HT, speculating that the presence of viral infection or inflammation could damage graft status. Assuming that the presence of cell inflammation at EMB could be a sign of future complete LV recovery, an accurate diagnosis of acute myocarditis could legitimize the continuation of medical intervention, delaying listing for HT [44].

The detection of viral pathogens in myocardial samples could be useful for a pathogen-tailored treatment strategy. There are no published evidence-based data on pathogen-specific therapy for viral myocarditis, but according to the protocols that are implemented for adult studies, specific treatments have been proposed. 

The exclusion of viral persistence during EMB is mandatory for the use of immunosuppressive therapy since it is strongly contraindicated in enterovirus- and adenovirus-positive patients. 

Frustaci et al. [45] reported the results of a treatment with prednisone and azathioprine in patients with biopsy-proven myocarditis for 6 months. Half of these patients showed an increase in LV function that ranged from 23 to 47%. Cardiac antibodies were present in almost all the responders, while the viral genome at EMB was only detected in non-responsive patients. Thus, the responsiveness to immunosuppressive therapy seems to be defined by the absence of viral genomes and the presence of immune upregulation. 

The same results were obtained in the Tailored Immunosuppression in Inflammatory Cardiomyopathy Study [46]: patients receiving immunosuppression showed a significant improvement in LVEF compared to those receiving placebo treatment.

Specific antiviral therapy in patients with virus-positive myocarditis has also been described although it has not been well established. The phase II BICC trial [47] studied the effect of immunomodulation with IFNβ therapy on viral load reduction in patients with myocarditis and myocardial viral presence (adenovirus, enterovirus, or parvovirus B19). Treatment with IFNβ was related to a reduction in the viral load in patients with adenovirus- or enterovirus-positive myocarditis but not in those with PVB19 myocarditis. The antiviral drugs pocapavir and pleconaril as well as IVIG therapy showed some efficacy in neonates with enteroviral myocarditis [48,49]. However, all of the authors stated that antiviral therapy is not yet an established therapy and that it must only be used in highly experienced centers.

## 7. EMB in Cardiomyopathies

Endomyocardial biopsy did not show a great deal of utility in the diagnosis of cardiomyopathy. Knowledge concerning the genetics and molecular biology of primary cardiomyopathies has made progress, and many of the diagnoses could be made by peripheral blood analysis. Moreover, if the myocardial disease is associated with skeletal muscle myopathy, EMB can be replaced by a skeletal muscle biopsy.

### 7.1. Dilated Cardiomyopathy (DCM)

In idiopathic/primary DCM, EMB diagnosis may play a primary role in excluding active myocarditis. Histology is nonspecific when diagnosing fibrosis, myocyte nuclear enlargement, and sarcoplasmatic degenerative changes, such as perinuclear halo and myocyte clearing or vacuolization [50]. Immunohistochemistry may be useful for the detection of muscle fiber abnormalities, but skeletal muscle biopsy may be easier. Familial DCM accounts for 30% of cases, and the diagnosis of familial forms cannot be pursued by histology or imaging [51].

### 7.2. Hypertrophic Cardiomyopathy (HCM)

EMB is not usually used for the clinical diagnosis of HCM since it is commonly based on noninvasive diagnostic tools. EMB may only be useful in selected cases where the purpose is to exclude infiltrative or storage diseases [52]. 

### 7.3. Mitochondrial Diseases and Other Inherited Storage Diseases

Mitochondrial cardiomyopathies (MICs) can be associated with multiple organ malfunction but may also be the only or the main aspect of respiratory chain dysfunction. The phenotype is usually hypertrophic and tends to present without obstruction. Usually, the diagnosis of MICs is based on a skeletal muscle biopsy, biochemical analysis, or genetic tests, and EMB may only be useful in for isolated forms [50] or when a skeletal muscle biopsy is not useful for diagnosis. 

In isolated MICs, it is necessary to store frozen myocardial samples in order to search for mitochondrial DNA mutations in the homogenate tissue.

Glycogen storage diseases (e.g., glycogenosis) and lysosomal storage diseases (e.g., Niemann–Pick disease, mucopolysaccharidosis, etc.) are systemic metabolic diseases that are characterized by deficiencies in various enzymes in the metabolic pathway and may be involved when the heart mimics a hypertrophic cardiomyopathy. Usually, skeletal muscle biopsy is used as diagnostic, but EMB with special stains in order to assess the nature of storage products may also be useful [50]. 

### 7.4. Anderson–Fabry Disease

Anderson–Fabry disease is an X-linked inborn metabolic defect that is characterized by an accumulation of glycosphingolipids and may cause left ventricular hypertrophy. During diagnosis, EMB may be very useful, particularly in patients who develop the disease later in life. Typical findings are dense concentric lamellar bodies that can be seen with electron microscopy [50].

### 7.5. Restrictive Cardiomyopathy (RCM)

RCM is a primary heart disease that is characterized by increased filling pressure in the ventricles in the presence of normal wall motion and systolic function. The etiology could be idiopathic, inflammatory, infiltrative, related to systemic diseases, or associated with mutations in genes encoding the sarcomeric contractile proteins. EMB is not very useful for a clear diagnosis of RCM since this disease has no definite histological characteristics. However, in selected cases, it can be used with the aim to exclude infiltrative or storage diseases [53] or to differentiate RCM from pericardial constriction [54].

### 7.6. Arrhythmogenic Right Ventricle Cardiomyopathy (ARVC)

ARVC is characterized by the progressive replacement of the ventricular myocardium with fibrofatty tissue. The septum is not commonly involved. There is not a single gold standard investigation for ARVC diagnosis since it derives from an assessment of major and minor diagnostic criteria [55].

EMB is rarely recommended and is only suggested in selected cases when other non-invasive or invasive procedures cannot provide a definite diagnosis. Moreover, in sporadic forms (not familial), it could be used in order to rule out phenocopies (i.e., myocarditis, sarcoid, etc.) [50].

## 8. Conclusions

In the diagnostic work up of children with systolic heart failure, EMB-derived histological, immunohistochemical, and molecular biological information are still essential prerequisites to provide a proper diagnosis of myocarditis and for the proper management of these patients.

In our experience, EMB results affected the clinical management of children with cardiomyopathy in 40% of cases.

We observed major adverse events, including cardiac perforation, in 10% of cases.

Given the high incidence of serious complications, particularly in small children, the risk–benefit ratio should be evaluated carefully. 

The use of other noninvasive tests such as MRI are helpful to exclude other forms of cardiomyopathy, allowing this invasive procedure to only be performed only in selected cases.

## Figures and Tables

**Table 1:** Characteristics of our population.

Characteristics	
Patients	29
BEM	30
Mean age at BEM (years)	7.3 ± 6
Mean weight (kg)	29 ± 19
Children < 1 year	5 (17%)
Children < 8 kg	2 (7%)
Onset	
Heart failure	24 (83%)
AV block	1
Pericarditis	2
Abdominal pain in COVID pt	1
Right Ventricular dysfunction	1

**Table 2:** EMB diagnosis.

Diagnosis at EBM	
Myocarditis	17 (57%)
Idiopathic DCM	9
Carnitine deficiency	1
Genetics positive for Laminopathy	1
Uhl disease	1

## Data Availability

Not applicable.
